# Supporting Canadian families of children with disabilities: unmet needs and service gaps

**DOI:** 10.3389/fpubh.2026.1754401

**Published:** 2026-02-19

**Authors:** K. Pozniak, A. Swain, M. Rodrigues, G. Currie, A. Doherty-Kirby, D. Grahovac, J. Lebsack, W. Campbell, C. Humphreys, S. Patterson, S. Raha, J. Whitley, O. Kraus de Camargo

**Affiliations:** 1 CanChild Centre for Childhood Disability Research, McMaster University, Hamilton, ON, Canada; 2 Department of Pediatrics, McMaster University, Hamilton, ON, Canada; 3 Department of Psychiatry, Queen's University, Kingston, ON, Canada; 4 School of Nursing and Midwifery, Mount Royal University, Calgary, AB, Canada; 5 School of Rehabilitation Science, McMaster University, Hamilton, ON, Canada; 6 Faculty of Education, University of Ottawa, Ottawa, ON, Canada

**Keywords:** caregiver experiences, childhood disability, community services, disability services, family support needs, financial need, health services

## Abstract

**Introduction:**

Children who live with disabilities and/or chronic (or complex) medical conditions, and their families, require appropriate supports to thrive and reach their full potential. In Canada, child and family wellbeing has declined over the past years (1,2).

**Methods:**

This article highlights the second phase of a sequential exploratory research study that identified what supports and services Canadian children living with disabilities/health conditions and their parents/caregivers need and want, now and into the future. This second phase consisted of surveys with 72 parents/caregivers of children and youth with disabilities and/or healthcare needs to learn whether their needs for services and supports in different domains of life - school, community, medical, recreation, family, and financial – were being met.

**Results:**

Over half of respondents (52.5%) reported at least one unmet need (defined as “need but not receiving” services), and 16% reported between 3 and 5 unmet needs. The greatest gaps were in recreation, financial, and family supports. Individuals who were receiving services and supports were for the most part satisfied with them. However, depending on the type of service, between 6%−42% of individuals indicated that they were dissatisfied with some aspects of service.

**Discussion:**

Findings from this study highlight the importance of adopting a larger systems approach of support that will coordinate and integrate the activities of sectors such as healthcare, education, and social services across the lifespan.

## Introduction

Children who live with disabilities and/or chronic (or complex) medical conditions, and their families, require appropriate supports to thrive and reach their full potential. In Canada, child and family wellbeing has declined over the past years (1, 2). Although children's and families' needs vary depending on the particularities of health conditions, geographic location, and the unique situation of each family, key themes pertaining to challenges and needs are consistently identified in the literature. These challenges include: an overall lack of supports; supports that are fragmented and fraught with bureaucracy; lack of information about available supports; uneven access to services based on geography; and financial challenges because of the high cost of services and inadequate financial supports (3–8). Supports that are frequently identified as important and needed include coordinated and comprehensive services for the entire family, assistance with navigating services, adequate financial supports, respite care, educational supports for the child, mental health supports, assistive equipment and devices (e.g., for mobility, communication), and opportunities for leisure, recreation and play (6, 9, 10). Families needs also evolve throughout the life course, as youth age out of school and the pediatric healthcare and social support systems, and transition into adult systems (10).

This article reports on the second phase of an exploratory sequential research study that identified what supports and services Canadian children and parents/caregivers need and want, now and into the future. In the first phase of the study, we qualitatively identified broad themes pertaining to family needs for support (11). In the second phase of the study, we surveyed caregivers of children with disabilities or chronic health conditions to learn about their experiences with services and supports in different domains of life: school, community, medical, recreation, family, and financial. We wanted to learn whether their needs for services and supports were being met, and how satisfied they were with different aspects of the supports they were receiving.

## Materials and methods

### Conceptual framework

Conceptually, this study is informed by the World Health Organization's International Classification of Functioning, Disability and Health (ICF) (World Health Organization 2001); specifically, its translation into the concept of the F-words for Child Development. The F-Words framework describes the various domains of life that influence children's health (family; friends; fun; functioning; fitness; and future), and the interrelationship between them (12). It provides a holistic lens to capture the multiple factors affecting children's and families' wellbeing and identifies intersections across various life contexts. Children with disabilities receive supports and services in many settings, including healthcare and education. This study explored the spectrum of families' needs across systems and sectors.

### Study design

This article reports on the second phase of an exploratory sequential research study, in which findings from the first qualitative phase informed the development of a survey. In the first phase of the study, we engaged youth using arts-based methods in which they completed a visual “time capsule” activity book about their experiences during and after COVID-19. Youth and their parents also participated separately in virtual interviews. The visual activity book and interviews captured broad themes related to children and families' needs for supports and services following the COVID-19 pandemic (11). Based on these findings, we developed a survey to identify specific gaps in the delivery of services. The study received ethics approval from our university's ethics board (#16645).

### Survey development

Our research team, comprising researchers from healthcare, rehabilitation, and education, as well as four parents of children/youth with disabilities, developed the survey iteratively. The survey was designed to complement Phase I qualitative findings pertaining to service needs and gaps with quantitative data. It asked caregivers about the services and supports that respondents in Phase I identified they were either receiving or wished to be receiving. These support areas included: school (e.g., academic support services); community (e.g., health-related therapies); medical (including at-home nursing or respite); recreation (e.g., sports and other leisure activities such as drama); family (including family therapy and support groups for caregivers); and financial (e.g., government grants). For example, we built in separate sections pertaining to family and financial supports because caregiver interviews frequently referenced lack of support for caregivers (including mental health) and lack of funding. Next, caregivers rated on a 7-point scale whether the (i) type; (ii) amount; and (iii) quality of the services they were receiving met their child's needs (1 = Strongly disagree, 7 = Strongly agree). We asked about these three separately because one of the insights derived from Phase I interviews was that while supports often exist “on paper”, in reality they do not meet the needs of children and families for a variety of reasons, including lack of fit with child's needs, inadequate amount/frequency, and overall poor quality of service delivery.

Caregivers also were asked to use a 7-point scale to rate the importance of supports that caregivers in Phase had identified as important to them (1 = Important to a very small extent, 7 = Important to a very great extent). Finally, caregivers were asked to rate on a 7-point scale their child's satisfaction with areas of life that were identified as important in Phase I youth and caregiver interviews (1 = Completely dissatisfied, 7 = Completely satisfied). The survey also included questions about caregivers' experiences with finding and coordinating services for their children (which caregivers in Phase I identified as an important barrier), and their experiences with service disruptions during the COVID pandemic. Each question had the option of adding a qualitative comment for participants who wished to elaborate on their responses (a complete list of survey questions is included in Appendix 1).

In addition to the survey, caregivers were also asked to provide demographic information about themselves and their children. This included information such as their age, relationship to child, household income, language(s) spoken at home, province of residence. Caregivers were also asked about the number of children they had and their ages. For each child, participants were asked to identify any functional concerns based on the tool “About my Child” (13). This tool asks caregivers to identify concerns in the following domains: mobility, community, seeing, hearing, learning/remembering, solving everyday problems, self-care, behavior, pain, sleep, and using hand/fingers.

### Recruitment

Parents/caregivers of children and youth with disabilities and/or healthcare needs were invited to participate in the study. Since our group embraces a non-categorical model of disability (14), participation was open to caregivers of any child with a disability or chronic or complex medical condition regardless of diagnosis. Because we were particularly interested in children's and families' experiences and needs as they related to healthcare and education sectors (and the intersections between these sectors), we engaged caregivers whose children were school-aged at the time of pandemic lockdowns in 2020. Thus, we recruited caregivers of children between the ages of 8 and 24, since children with disabilities can attend high school until the age of 21 in some Canadian provinces. Participants were recruited through our research center's social media networks (including our online newsletter, closed Facebook group for parents partnering in research, as well through the social media networks of our parent investigators) as well as through a market research company, Leger, who shared our recruitment information with their pool of panelists. Individuals who were interested in participating in the survey were directed to an online screening form via Research Electronic Data Capture (REDCap) to ensure they met eligibility criteria. The research coordinator reviewed their responses and followed up via email if additional clarification was needed. Participants whose eligibility was confirmed were e-mailed the link to the consent form and survey on REDCap.

### Data analysis

#### Software and data processing

All analyses were conducted using Stata SE version 17.0 (StataCorp, College Station, TX). Raw survey data were cleaned and prepared for analysis, including recoding variables and generating derived indicators, (e.g., unmet needs, child functional concerns - definitions provided below), to support interpretation and summarization.

#### Sample and variable preparation

A total of 83 participants initiated the survey. Eleven participants were excluded due to negligible completion (i.e., they started the survey but answered only a few questions). The final analytic sample included 72 respondents who completed the survey. For ordinal variables with categorical responses such as “Not applicable” (coded as 8) and “Prefer not to answer” (coded as 9), values were recoded as missing prior to analysis.

New variables were derived as follows:

- **Unmet Needs Count**: A summary score was calculated based on the number of domains in which participants reported unmet needs (by checking “need but not receiving” services), including school, medical, recreational, family, financial and community-based support. A binary indicator was also created to capture the presence of *any* unmet need.
- **Children with Functional Concerns**: A derived count variable captured the total number of children in the household identified as having functional concerns on the About my Child questionnaire (question #14).

### Descriptive analysis

Frequencies and percentages were used to summarize categorical variables. Medians and interquartile ranges (IQR) were reported for ordinal variables, while means and standard deviations were used for continuous variables (e.g., age), where appropriate.

Summary tables were generated using Stata's table1 command. Where applicable, comparisons were stratified by subgroups (e.g., by financial support or receipt of services). Chi-squared or t-tests were used for comparisons across two groups (e.g., received vs. not received; unmet vs. no unmet needs; interrupted vs. not interrupted) within each domain: school supports, community supports, recreational supports, medical supports, family supports, unmet needs, finances received, interrupted services. When comparing across three or more groups (e.g., received, not received, unmet need), one-way ANOVA was used if all categories included at least 10 observations; otherwise, categories were collapsed into the aforementioned binary groupings.

Analytical decisions were guided by distributional characteristics and subgroup cell sizes, consistent with the exploratory nature of the study, rather than formal modeling assumptions. Ordinal and skewed variables were summarized using non-parametric approaches, and categories were collapsed where counts were sparse to ensure stable and interpretable estimates.

### Correlation analysis

Descriptive pairwise correlations were conducted using Kendall's tau-b, a non-parametric measure suitable for ordinal and skewed continuous variables. Correlations were estimated between selected variables, including age, household income, number of children with concerns, financial indicators, and satisfaction within each domain.

Statistical significance was set at the 0.05 level; however, all analyses, including subgroup comparisons, were exploratory and descriptive in nature. Results were interpreted descriptively, as no formal hypothesis testing, multivariable modeling, or adjustment for potential confounding was conducted. Accordingly, *p*-values are reported for descriptive context only and should not be interpreted as confirmatory evidence.

#### Analysis of qualitative survey comments

Respondents' qualitative comments pertaining to each domain (e.g., school, community, medical supports) were analyzed using qualitative description (15). This approach allows researchers to stay close to the data in order to document and describe participants' perspectives on their own experience without reinterpretation.

#### Integration of quantitative and qualitative data from surveys

**Figure 1:**
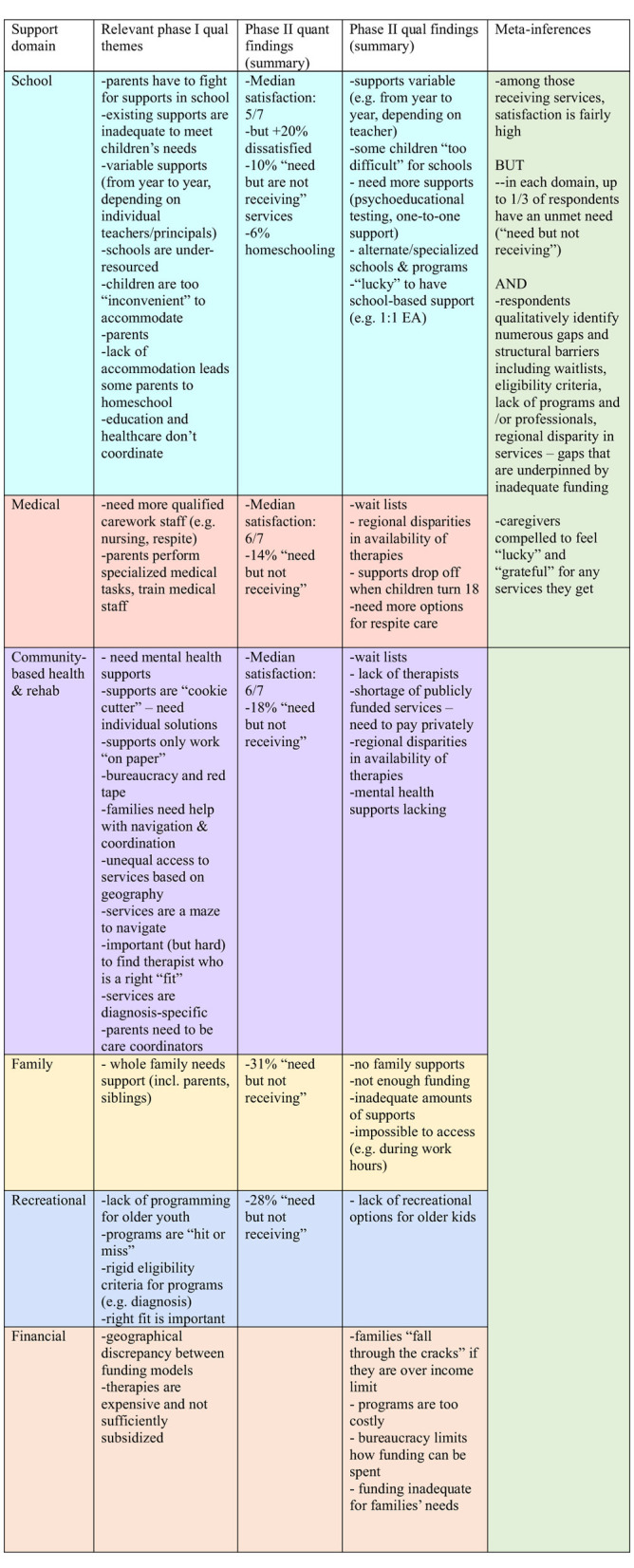
Data integration map.

We used a weaving approach (16), in which quantitative and qualitative findings are brought together on a theme-by-theme or concept-by-concept basis. We compared quantitative and qualitative findings to identify convergence, complementarity and dissonance (17, 18). At this stage we noted that there was confirmation between qualitative insights derived from Phases I and II, but also contradiction between quantitative and qualitative findings. By integrating insights from these two sets of data, we developed metainferences to explain these phenomena (see Figure 1 for our Data Integration Map).

#### Respondent characteristics

A total of 72 responses were included in the final analysis. Fifty-seven percent of caregivers identified as mothers, 35% as fathers, 3% as grandmothers, and 6% (*n* = 4) opted to self-identify (responses included “birthing parent”, “legal guardian and maternal aunt”, “sibling” and “non-gendered parent”). Respondents lived in eight Canadian provinces, and their mean age was 45.2 years (*SD* = 8.9). Eighty-five per cent (*n* = 65) of respondents were born in Canada. Forty percent of caregivers had at least one child in their household, 42% had two children, 11% had three children, and 1% had four and five children, respectively. The mean age of the children was 13.28 years (*SD* = 4.41) with a range from less than 1 to over 18 years of age. Caregivers reported their children as presenting a wide range of impairments and restrictions, with many reporting multiple concerns. The most commonly reported were: learning/remembering (76%), solving everyday problems (52%), self-care (50%), behavior (48%) and communicating (44%). Caregivers also qualitatively added additional concerns, including diet/restrictive food choices, anxiety, digestive symptoms, and mental health. Because our group embraces a non-diagnostic model of disability, caregivers were not asked to provide their children's diagnosis. Nonetheless, several respondents mentioned their children's diagnoses in their qualitative comments. These included autism, Down Syndrome, Tourette's syndrome, dyslexia and dyspraxia. More comprehensive participant demographic information can be found in Table 1.

**Table 1:** Demographic data.

**07.07.2025**	***N* = 72**
Participants' age	Mean = 45.17 (Min = 26, max = 67, *SD* = 8.9)
Participant's sex	Female, *n* = 44 Male, *n* = 25 Other, *n* = 3
Participant's relationship to a child	Mother, *n* = 41 Father, *n* = 25 Grandmother, *n* = 2 Other, *n* = 4 (sibling, birthing parent, non-binary parent, legal guardian (maternal aunt))
Province	Alberta, *n* = 9 British Columbia, *n* = 12 Manitoba, *n* = 4 Nova Scotia, *n* = 3 Ontario, *n* = 32 PEI, *n* = 2 Quebec, *n* = 7 Saskatchewan, *n* = 3
Community	Large city/urban area (>500,000 people), *n* = 35 Small or medium sized town (100,000-500,000 people), *n* = 21 Rural area, *n* = 13 Other, *n* = 3 (small city)
Level of education	Completed high school, *n* = 3 Some college or university, *n* = 10 Completed college or university, *n* = 43 Graduate school, *n* = 5 Postgraduate/professional degree, *n* = 11
Current work situation (Select all that apply)	Employed full-time, *n* = 37 Employed part-time, *n* = 16 Not in the paid workforce at this stage of my life, *n* = 5 Volunteering, *n* = 1 Full-time caregiver, *n*= 6 Self-employed, *n* = 9 Other, *n* = 6 (multiple part-time jobs, full-time student / continue education courses while employed, retired)
English is a first language	*N* = 59
Language(s) spoken at home (select all that apply)	English, *n* = 70 French, *n* = 14 Indigenous language, *n* = 2 Not listed here/prefer to self-describe, *n* = 4 (Urdu, Greek, Hebrew, Danish)
Birth country	Canada, *n* = 61 Mali, *n* = 2 Pakistan, *n* = 1 India, *n* = 3 Mexico, *n* = 1 Kenya, *n* = 1 Liberia, *n* = 1 Hong Kong, *n* = 1 New Zealand, *n* = 1
Years lived in Canada, if immigrated	0-5 years, *n* = 2 10+ years, *n* = 9
Access to government-funded healthcare (e.g., OHIP)?	Yes, *n* = 66 No, *n* = 6
Ethnicity, self-identified (Select all that apply)	Indigenous (First Nations/Metis/Inuit), *n* = 5 South Asian (e.g., East Indian, Pakistani, Sri Lankan, etc.), *n* = 6 Chinese, *n* = 4 Black, *n* = 5 Filipino, *n* = 1 Latin American, *n* = 1 Arab, *n* = 2 White/Caucasian, *n* = 47 Not listed here/prefer to self-describe, *n* = 3 (Biracial, Irish, Taiwanese or East Asian) Prefer not to answer, *n* = 1
Total household income	< $25K, *n* = 1 $25- < 50K, n = 8 $50- < 70K, *n* = 12 $70 < 100K, *n* = 16 $100-150K, *n* = 20 >$150K, *n* = 13 Prefer not to answer, *n* = 2
Number of children at home	1. child, *n* = 29 (40%) 2. children, *n* = 30 (42%) 3. children, *n* = 11 (15%) 4. children and more, *n* = 2 (2%)
Age of all children at home	Mean = 13.28 (Min = 1, Max = 20, *SD* = 4.41)
Concerns for all children living at home (select all that apply)	• Mobility (walking, crawling, getting around), *n* = 8 • Communicating, *n* = 43 • Seeing, *n* = 3 • Hearing, *n* = 6 • Learning/remembering, *n* = 60 • Solving everyday problems, *n* = 41 • Self-care (such as feeding, dressing, using the toilet), *n* = 28 • Behaviour, *n* = 49 • Pain, *n* = 11 • Sleep, *n* = 27 • Using hands/fingers, *n* = 10 • No concerns, *n* = 35 • Not listed here/prefer to self-describe, *n* = 16 • Prefer not to answer, *n* = 1
Number of other adults live in participants' homes	The only adult, *n* = 7 2 adults, *n* = 46 3 adults, *n* = 17 4 adults, *n* = 2
Other adults' relationships to participants' child(ren) (textbox)	Mother, *n* = 19 Father, *n* = 31 (Biological) parent(s), *n* = 5 Partner, spouse, step-parent, wife, husband, *n* = 8 Sibling(s), *n* = 6 Grandparent(s), *n* = 4 Other, *n* = 2 (aunt)
Other significant caregivers (textbox)	No one/the only caregiver, *n* = 11 Child(ren)'s mother, *n* = 2 Child(ren)'s father, *n* = 12 Husband, wife, spouse, (ex-)partner, step-parent, *n* = 16 Grandparent(s), *n* = 17 Sibling(s), older or younger, *n* = 3 Extended family (aunt, uncle), *n* = 3 Close friends, n = 2 School staff, hospital staff, *n* = 2

## Results

**Figure 2:**
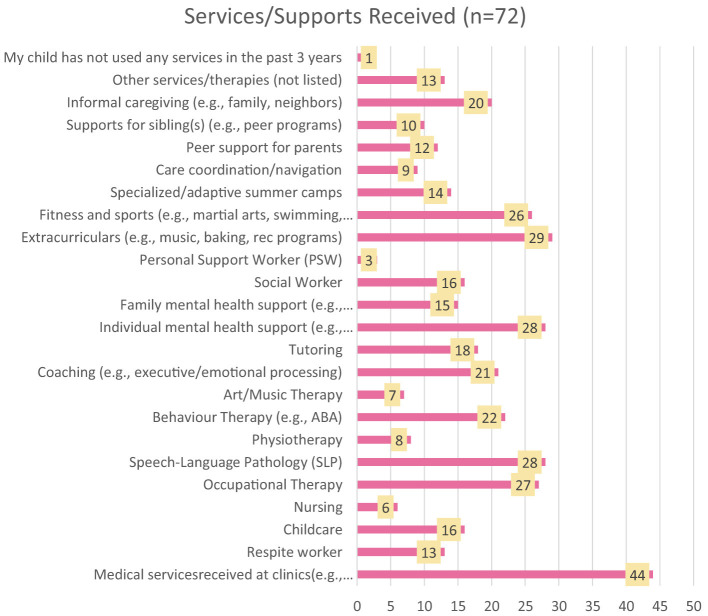
Supports that families were receiving.

In all, participants reported accessing over 20 different services and supports in the past three years, with only 1 respondent indicating that their child had not accessed any. The most frequently accessed supports were extracurricular activities (40%), individual mental health supports (39%), speech therapy (39%), occupational therapy (38%), fitness and sports (36%), and behavior therapy (31%). A complete list of supports and services accessed by respondents is included in Figure 2.

In each domain (school, community, medical, recreation, family, and financial), between 10-31% of respondents indicated they had an unmet need (i.e., “need but not receiving” services). The highest unmet needs were in the area of family supports (31%) and recreation (28%). When unmet needs were aggregated across domains, 52.5% respondents (*n* = 38) reported at least 1 unmet need; 16% (*n* = 12) reported between 3 to 5 unmet needs. On the whole, 74% of female respondents versus 18% of male respondents identified at least one unmet need (defined as “needs but is not receiving” a particular type of service, *p* = 0.004). Respondents who identified as sole caregivers were also somewhat more likely to identify having an unmet need (*p* = 0.082). For the most part, individuals who were receiving services and supports reported that these supports met their needs. However, depending on the area, between 6-42% of individuals indicated that some aspects of the service did not meet their needs. In what follows we provide a more in-depth look into caregivers' experiences with the services their families were receiving.

### School-based supports

The majority (76% or *n* = 55) of caregivers indicated that their children were receiving services at school, whereas 10% (*n* = 7) of caregivers reported that their children needed, but were not receiving, services. An additional three caregivers reported that their children were homeschooled, which they explained was due to lack of needed supports at school. One caregiver wrote: “My son has been formally kicked out of school without any plan in place because nobody can handle him”. Caregivers whose children were receiving services reported that these services for the most part met their children's needs (mean 5/7). However, 16% reported that the type of service, 20% that the quality, and 26% that the amount, did not meet their children's needs. Caregivers' qualitative comments indicated that the services and supports were variable and contingent on factors such as having dedicated and invested staff. When asked about specific supports that would have been helpful, caregivers mentioned after-school programs that could accommodate children's needs and specific school-based supports (e.g., psychoeducational testing, more one-to-one support).

### Medical supports

Sixty percent of the respondents indicated that their children were receiving medical and caregiving supports (including nursing care), with 14% indicating that their child needed medical supports but was not receiving them. Among those receiving services, 11% indicated their child was not receiving the appropriate type of service, 13% the amount, and 6% the quality. Caregivers' comments referenced the shortage of needed supports (including therapies, medical testing and respite), long wait lists, costs of therapies that are delivered privately: “My child needs occupational therapy to help with dyspraxia but there aren't enough OTs in my city and I don't receive enough autism funding to cover the cost of her autism therapies much less the other therapies she needs”.

### Community-based health and therapy supports

Fifty-three percent of respondents indicated that their child was receiving community-based health and therapy supports, with 18% indicating that their child needed these supports but was not receiving them. Eleven percent of respondents were dissatisfied with the type, 16% with the amount, and 8% with the quality of supports they received. As with medical services, caregivers' comments referenced the limited services available, and the long wait lists and problems of access: “There isn't one person in [city] that I could find through calling or emailing any services I could find for help with Dyslexia and Dysgraphia”; “On the waiting list at the local [organization] for years”.

### Family supports

Thirty-nine percent of caregivers reported that they were receiving family supports, and 31% indicated that they needed but were not receiving them. Caregivers' comments referenced the overall lack of supports for caregivers, for example: “There is no funding to help nor support caregivers”. They also highlighted the high cost of services, and the decline of services for caregivers as children age: “There are no parental supports for families who have adult children”.

### Recreational supports

With regards to recreational activities, 61% of respondents (*n* = 44) indicated that their child was participating in recreational activities. However, 22% of respondents indicated that their child would like to participate, but existing activities were not accommodating their needs, and 6% indicated that recreational activities were not available in their community. One caregiver noted that recreational opportunities declined as children age: “Unfortunately there aren't a lot of summer camps and day camps and recreation for special needs kids over 12 [years].”

### Financial supports

More than half (60%, *n* = 43) of respondents indicated that they had applied for government financial supports in the past three years. Of these, 79% (*n* = 33) indicated that they had received this support. Thirteen percent of respondents (*n* = 9) indicated that they had applied for but had not received financial support. Forty-two percent of individuals who received financial support reported that the amount did not meet their needs, and 27% of recipients indicated that they were not able to spend it in a way that best met their family's needs. Participants provided extensive comments regarding the high cost of therapies, programs and equipment, and the low levels of available funding: “[Son] gets a pithy $350/month for respite. We pay over $1200/month for his classes at Down Syndrome Resource Centre. As you can see, the government money does not come even close to covering it”.

Participants who were not receiving funding commented that eligibility thresholds disqualified many families: “My child needs a speech language pathologist and a psychoeducational evaluation, but each costs hundreds or thousands of dollars that we do not have as a lower middle class family. Even with his genetic condition and congenital heart defect, he does not qualify for help from the government because of budget cuts”.

Some participants also noted the bureaucratic hoops that limited their ability to use funding in a way that best worked for their family: “It's easy to get the financial support but hard to spend it. You have to jump through a lot of hoops and can only spend it on what they think is appropriate”.

### What families need to be at their best

In the first initial qualitative phase of this study, youth and parents identified what would enable their families to thrive. These included: continuous services (no ageing out); invested service providers who were a good fit for the child and family; consistent and reliable communication between school personnel and healthcare professionals/therapists; services/therapies that are available, affordable, and easy to navigate; assistance with navigating services; recreation activities that meet the child's needs; having the option of remote services, schooling and medical appointments; and virtual connections with other children and caregivers (11). In this second phase, we asked caregivers to rate the importance of these supports on a scale of 1 (not important at all) to 7 (very important). All of the above-mentioned supports were rated as important, with median scores between 6 and 7 on almost every item except for the two “virtual connections” items, which were both rated 5. When asked in an open-ended format what other supports their child and family would need to thrive, caregivers repeatedly listed mental health supports for the entire family, respite care, attention to the needs and wellbeing of caregivers, and better financial supports. Caregivers also mentioned specific therapies and equipment, school-based supports, and recreational supports.

## Discussion

### Support needs and gaps

This survey adopted a holistic lens to capture high-level patterns in families' unmet needs and satisfaction with services and supports. On the whole, caregivers identified the greatest gaps in recreation, financial, and family supports, rather than in the healthcare system. This finding confirmed the importance of adopting a holistic perspective to child health and wellbeing – one that focuses on areas of life that are important to children themselves (e.g., recreational activities in which they can take part), as well as on the needs of the entire family, including parents/caregivers. Parents' comments on the survey also illustrate that many of the issues related to service delivery are systemic and intertwined. For example, due to overall lack of services there are long wait lists. The costs of available services are high relative to the financial supports provided to families, impacting families' access. At the policy level, this highlights the importance of adopting a larger systems approach that will coordinate the activities of sectors such as healthcare, education, and social services.

Parents' comments also illustrate the decline of disability-related supports as children age and the paucity of services of adults. There is a need to better integrate systems of supports across the lifespan, and improve services for adults. Relevant supports for adults can include appropriate funding for programs and equipment, day programs and extracurricular activities, and continuity of care from pediatric to adult services, along with training of healthcare providers so they can support adults with childhood-onset health conditions. These findings align with recommendations made by other consortiums on child health such as Children's Healthcare Canada (2) as well as Canada's Disability Inclusion Action Plan (19).

When asked to rate the importance of a list of supports identified by parents in the previous qualitative phase as important, the caregivers in this phase rated all of them as important (with median scores between 6 and 7 out of 7 on almost every item). This further validates their importance, as well as aligns with existing literature in the area which underscores the importance of services that are continuous, coordinated (both within and across sectors), and accessible, and easy to navigate (4, 10, 20). Prominent among the specific supports that parents identified in their qualitative comments as lacking were caregiving supports and mental health supports for the entire family. These findings align with the work of many scholars and organizations in this area, who have drawn attention to the high burden associated with caregiving (21–23) and the high rates of mental health issues among Canadians (24) Caregivers also repeatedly mentioned financial barriers (including the high cost of services and inadequate financial supports, a fact also recognized in the literature (4, 25). For example, it has been reported that the majority of people who are eligible for disability-related supports do not access them, due to factors such as lack of information about available programs, difficulties in completing the application process, prohibitive eligibility criteria (e.g., regarding type or severity of diagnosis), and inaccessible program design/delivery (e.g., length of waitlists and program duration (4).

These findings indicate that programs and policies regarding mental health, caregiving and disability-related financial supports need to be re-evaluated as well as better integrated to acknowledge their interrelated nature.

### Discrepancy between quantitative scores and qualitative comments

On the whole, caregivers' satisfaction scores across services were fairly high. This suggests that those caregivers who are receiving services are for the most part fairly content with them. Nonetheless, half of respondents had at least one unmet need for services and supports, and existing supports did not meet the needs of between 20%−40% of their recipients (depending on the type of support). Furthermore, parents' comments overwhelmingly referenced shortcomings and challenges such as wait times and inadequate supports. These qualitative comments echoed the issues noted by parents in the first (interview) phase of this study (11). They also align with other qualitative studies that identify similar issues in the experiences of caregivers of children with disabilities and/or medical complexities in Canada (26, 27). Therefore, there is a discrepancy between the overall positive survey ratings and caregivers' qualitative reports. A similar phenomenon was also noted by other researchers (28, 29) in the context of patients' feedback on their hospital admissions. In what follows we offer several interpretative hypotheses that may explain this contradiction and its possible implications.

One possible explanation for overall positive ratings is that within an overall model of service scarcity, caregivers become accustomed to viewing scarcity as the norm, and therefore appreciate any long-awaited services they do receive. In the qualitative comments, as well as in the interviews done as part of the previous phase of this study, several caregivers described themselves as “lucky” to be receiving services, and expressed feeling “grateful” for these services. This could explain the relatively high satisfaction ratings among those respondents who were receiving services.

Another possible explanation is that we did not require that the respondents be primary caregivers. Primary caregivers may be more likely to be aware of unmet needs and limits of services, as they are the ones who are primarily responsible for navigating these services. Therefore, the scores across various domains might have been different (possibly lower) if we had surveyed primary caregivers only.

In all, we suggest that the scores and the qualitative comments taken together provide a more accurate and nuanced picture of caregivers' experiences than each do on their own. These experiences appear to be checkered: families are able to access some needed supports but not others.

### Limitations

This study provides a holistic, high-level, picture of family needs related to services and supports for their children. While subgroup differences were observed, these findings are exploratory and hypothesis-generating and should not be interpreted as evidence of causation or confirmatory statistical inference. Rather, they offer insight into patterns that warrant further investigation in larger, hypothesis-driven studies. In addition, the survey did not delve into specifics of services (for example, whether medical/healthcare services were provided in a family-centered way). Therefore, we have limited information regarding the qualities that make various services desirable to families.

Another limitation of this study is the demographic composition of our sample. Most participants were Canadian-born, identified as White, spoke English as their first language, and had at least a university or college-level education. As a result, the study underrepresents families who face cultural, socioeconomic, and other structural barriers. This lack of representation likely influenced the findings, as the number of unmet needs and levels of dissatisfaction with services may have been higher had families from underserved populations been more adequately represented.

### Future research

Additional research is needed to address experiences with service delivery and unmet needs among equity-deserving groups specifically, including newcomers to Canada, Indigenous populations, and those living in rural communities. Furthermore, studies with a larger sample size would support comparisons between subgroups.

## Conclusion

Children with disabilities and their families require holistic supports across all domains of life. However, caregivers reported that some of their child and family's needs were not consistently being met. In particular, caregivers reported unmet needs related to recreation, services for caregivers, and financial supports. These findings illustrate the need to adopt a holistic perspective to child health and disability that considers opportunities for play and participation, and see the child as part of a larger family unit that also needs to be supported. There is an urgent need for policymakers to act on these insights to implement comprehensive, family-centered solutions that will address these gaps through sustained investment and cross-sector collaboration.

## Data Availability

The datasets presented in this article are not readily available in order to protect participants' confidentiality. Requests to access the datasets should be directed to pozniakk@mcmaster.ca; krausdc@mcmaster.ca.
