# Supplementary material for: Supporting Canadian families of children with disabilities: unmet needs and service gaps
Source: Front Public Health. 2026 Feb 19;14:1754401. doi: 10.3389/fpubh.2026.1754401 (PMC12960534; doi:10.3389/fpubh.2026.1754401)
Supplement: Supplementary file 1 [file Data_Sheet_1.pdf]

Time Capsule Survey

Note: If you would like to turn on the Text-To-Speech functionality (text on the page will be read audibly to you), please click on the sound icon at the top right of this page to enable this function. Please note that for longer sections of text, the sound icon to play the audio may be towards the bottom of the section.

You can also adjust the font size by clicking the + and - icons next to the sound icon.

Please enter your study ID (provided to you by research coordinator) __________________________________

Therapies and Supports

---

1 What services and supports has your child(ren) received and/or taken part in during the past 3 years? Check all that apply.

- ☐ Nursing
- ☐ Occupational Therapy (OT)
- ☐ Speech-Language Pathology (SLP) services
- ☐ Physiotherapy
- ☐ Behaviour therapy (e.g., ABA)
- ☐ Art/music therapy
- ☐ Coaching of particular personal or social skills (e.g., executive functioning, emotion processing)
- ☐ Tutoring
- ☐ Individual mental health supports (individual counselling or psychotherapy for your child/ren)
- ☐ Family mental health supports (couple/family counselling or psychotherapy)
- ☐ Social worker
- ☐ Personal Support Worker (PSW)
- ☐ Extracurriculars (e.g., arts, music, baking class, library programs)
- ☐ Fitness and sports (e.g., martial arts, swimming, dancing, horseback riding)
- ☐ Specialized/accommodated summer camps
- ☐ Care coordination, service navigation
- ☐ Peer support groups for parents
- ☐ Supports for your child's sibling(s) (e.g., caregiving, peer support, etc.)
- ☐ Informal caregiving supports (from friends, family members, neighbors)
- ☐ Other therapies/services/supports not mentioned here
- ☐ My child hasn't received any services and supports in the past 3 years
- ☐ Prefer not to answer

(Check all that apply)

Please describe ____

Where does your child(ren)/family receive nursing services? Please check all that apply.

- ☐ At school
- ☐ At home
- ☐ In the community (e.g., through a public or private organization)
- ☐ In hospitals or medical clinics
- ☐ Other
- ☐ Prefer not to answer

Please describe ____

How were/are nursing services being delivered?	☐ Online<br>☐ In-person<br>☐ Hybrid (a combination of online and in-person)<br>☐ Prefer not to answer
Where does your child(ren)/family receive Occupational Therapy (OT) services? Please check all that apply.	☐ At school<br>☐ At home<br>☐ In the community (e.g., through a public or private organization)<br>☐ In hospitals or medical clinics<br>☐ Other<br>☐ Prefer not to answer
Please describe	__________________________________
How were/are occupational therapy (OT) services being delivered?	☐ Online<br>☐ In-person<br>☐ Hybrid (a combination of online and in-person)<br>☐ Prefer not to answer
Where does your child(ren)/family receive Speech-Language Pathology (SLP) services? Please check all that apply.	☐ At school<br>☐ At home<br>☐ In the community (e.g., through a public or private organization)<br>☐ In hospitals or medical clinics<br>☐ Other<br>☐ Prefer not to answer
Please describe	__________________________________
How were/are Speech-Language Pathology (SLP) services being delivered?	☐ Online<br>☐ In-person<br>☐ Hybrid (a combination of online and in-person)<br>☐ Prefer not to answer
Where does your child(ren)/family receive Physiotherapy services? Please check all that apply.	☐ At school<br>☐ At home<br>☐ In the community (e.g., through a public or private organization)<br>☐ In hospitals or medical clinics<br>☐ Other<br>☐ Prefer not to answer
Please describe	__________________________________
How were/are Physiotherapy services being delivered?	☐ Online<br>☐ In-person<br>☐ Hybrid (a combination of online and in-person)<br>☐ Prefer not to answer

Where does your child(ren)/family receive Behaviour therapy (e.g., ABA) services? Please check all that apply.	☐ At school<br>☐ At home<br>☐ In the community (e.g., through a public or private organization)<br>☐ In hospitals or medical clinics<br>☐ Other<br>☐ Prefer not to answer
Please describe	______________________________
How were/are Behaviour therapy (e.g., ABA) services being delivered?	☐ Online<br>☐ In-person<br>☐ Hybrid (a combination of online and in-person)<br>☐ Prefer not to answer
Where does your child(ren)/family receive Art/music therapy services? Please check all that apply.	☐ At school<br>☐ At home<br>☐ In the community (e.g., through a public or private organization)<br>☐ In hospitals or medical clinics<br>☐ Other<br>☐ Prefer not to answer
Please describe	______________________________
How were/are Art/music therapy services being delivered?	☐ Online<br>☐ In-person<br>☐ Hybrid (a combination of online and in-person)<br>☐ Prefer not to answer
Where does your child(ren)/family receive Coaching of particular personal or social skills (e.g., executive functioning, emotion processing) services? Please check all that apply.	☐ At school<br>☐ At home<br>☐ In the community (e.g., through a public or private organization)<br>☐ In hospitals or medical clinics<br>☐ Other<br>☐ Prefer not to answer
Please describe	______________________________
How were/are Coaching of particular personal or social skills services being delivered?	☐ Online<br>☐ In-person<br>☐ Hybrid (a combination of online and in-person)<br>☐ Prefer not to answer
Where does your child(ren)/family receive Tutoring services? Please check all that apply.	☐ At school<br>☐ At home<br>☐ In the community (e.g., through a public or private organization)<br>☐ In hospitals or medical clinics<br>☐ Other<br>☐ Prefer not to answer

Please describe	__________________________________
How were/are Tutoring services being delivered?	☐ Online<br>☐ In-person<br>☐ Hybrid (a combination of online and in-person)<br>☐ Prefer not to answer
Where does your child(ren)/family receive Individual mental health supports (individual counselling or psychotherapy for your child/ren) services? Please check all that apply.	☐ At school<br>☐ At home<br>☐ In the community (e.g., through a public or private organization)<br>☐ In hospitals or medical clinics<br>☐ Other<br>☐ Prefer not to answer
Please describe	__________________________________
How were/are Individual mental health support services being delivered?	☐ Online<br>☐ In-person<br>☐ Hybrid (a combination of online and in-person)<br>☐ Prefer not to answer
Where does your child(ren)/family receive Family mental health supports (couple/family counselling or psychotherapy) services? Please check all that apply.	☐ At school<br>☐ At home<br>☐ In the community (e.g., through a public or private organization)<br>☐ In hospitals or medical clinics<br>☐ Other<br>☐ Prefer not to answer
Please describe	__________________________________
How were/are Family mental health support services being delivered?	☐ Online<br>☐ In-person<br>☐ Hybrid (a combination of online and in-person)<br>☐ Prefer not to answer
Where does your child(ren)/family receive Social work services? Please check all that apply.	☐ At school<br>☐ At home<br>☐ In the community (e.g., through a public or private organization)<br>☐ In hospitals or medical clinics<br>☐ Other<br>☐ Prefer not to answer
Please describe	__________________________________
How were/are Social work services being delivered?	☐ Online<br>☐ In-person<br>☐ Hybrid (a combination of online and in-person)<br>☐ Prefer not to answer

Where does your child(ren)/family receive Personal Support Worker's (PSW) services? Please check all that apply.	☐ At school<br>☐ At home<br>☐ In the community (e.g., through a public or private organization)<br>☐ In hospitals or medical clinics<br>☐ Other<br>☐ Prefer not to answer
Please describe	__________________________________
How were/are Personal Support Worker (PSW) services being delivered?	☐ Online<br>☐ In-person<br>☐ Hybrid (a combination of online and in-person)<br>☐ Prefer not to answer
Where does your child(ren)/family receive Extracurriculars (e.g., arts, music, baking class, library programs) services? Please check all that apply.	☐ At school<br>☐ At home<br>☐ In the community (e.g., through a public or private organization)<br>☐ In hospitals or medical clinics<br>☐ Other<br>☐ Prefer not to answer
Please describe	__________________________________
How were/are Extracurriculars being delivered?	☐ Online<br>☐ In-person<br>☐ Hybrid (a combination of online and in-person)<br>☐ Prefer not to answer
Where does your child(ren)/family receive Fitness and sports (e.g., martial arts, swimming, dancing, horseback riding) services? Please check all that apply.	☐ At school<br>☐ At home<br>☐ In the community (e.g., through a public or private organization)<br>☐ In hospitals or medical clinics<br>☐ Other<br>☐ Prefer not to answer
Please describe	__________________________________
How were/are Fitness and sports being delivered?	☐ Online<br>☐ In-person<br>☐ Hybrid (a combination of online and in-person)<br>☐ Prefer not to answer
Where does your child(ren)/family receiving Specialized/accommodated summer camps services? Please check all that apply.	☐ At school<br>☐ At home<br>☐ In the community (e.g., through a public or private organization)<br>☐ In hospitals or medical clinics<br>☐ Other<br>☐ Prefer not to answer

Please describe

____

How were/are Specialized/accommodated summer camps services being delivered?

- ☐ Online
- ☐ In-person
- ☐ Hybrid (a combination of online and in-person)
- ☐ Prefer not to answer

Where does your child(ren)/family receive Care coordination and/or service navigation? Please check all that apply.

- ☐ At school
- ☐ At home
- ☐ In the community (e.g., through a public or private organization)
- ☐ In hospitals or medical clinics
- ☐ Other
- ☐ Prefer not to answer

Please describe

____

How were/are Care coordination and/or service navigation services being delivered?

- ☐ Online
- ☐ In-person
- ☐ Hybrid (a combination of online and in-person)
- ☐ Prefer not to answer

Where does your child(ren)/family receive Peer support groups for parents services? Please check all that apply.

- ☐ At school
- ☐ At home
- ☐ In the community (e.g., through a public or private organization)
- ☐ In hospitals or medical clinics
- ☐ Other
- ☐ Prefer not to answer

Please describe

____

How were/are Peer support groups for parents being delivered?

- ☐ Online
- ☐ In-person
- ☐ Hybrid (a combination of online and in-person)
- ☐ Prefer not to answer

Where does your child(ren)/family receive Supports for your child's sibling(s) (e.g., caregiving, peer support, etc.) services? Please check all that apply.

- ☐ At school
- ☐ At home
- ☐ In the community (e.g., through a public or private organization)
- ☐ In hospitals or medical clinics
- ☐ Other
- ☐ Prefer not to answer

Please describe

____

How were/are supports for your child(ren)'s sibling(s) being delivered?

- ☐ Online
- ☐ In-person
- ☐ Hybrid (a combination of online and in-person)
- ☐ Prefer not to answer

Where does your child(ren)/family receive Informal caregiving supports (from friends, family members, neighbors)? Please check all that apply.

- ☐ At school
- ☐ At home
- ☐ In the community (e.g., through a public or private organization)
- ☐ In hospitals or medical clinics
- ☐ Other
- ☐ Prefer not to answer

Please describe

____

How were/are informal caregiving supports being delivered?

- ☐ Online
- ☐ In-person
- ☐ Hybrid (a combination of online and in-person)
- ☐ Prefer not to answer

Where does your child(ren)/family receive Other services (not listed above)? Please check all that apply.

- ☐ At school
- ☐ At home
- ☐ In the community (e.g., through a public or private organization)
- ☐ In hospitals or medical clinics
- ☐ Other
- ☐ Prefer not to answer

Please describe

____

How were/are other (not listed above) services being delivered?

- ☐ Online
- ☐ In-person
- ☐ Hybrid (a combination of online and in-person)
- ☐ Prefer not to answer

Supports at School

2 What services and supports has your child(ren) received and/or taken part in during the past 3 years? Check all that apply.

- ☐ Access to Education Assistant (EA)
- ☐ Resource teacher
- ☐ Individualized Education Plan (IEP, PLP)
- ☐ Accessible school environment (e.g., accessible physical space and/or transportation, available interpreter, child's integration in the classroom, etc.)
- ☐ Teachers who understand and accommodate my child's needs
- ☐ Tier 2/3 intervention programs (e.g., an intensive literacy program)
- ☐ Other supports at school not listed here
- ☐ My child hasn't received any supports in school in the past 3 years
- ☐ Prefer not to answer

Please describe:

____

3 Tell us more about school-based services and supports (e.g., resource teacher, school nurse) your child(ren) has received in the past 3 years.

- ◯ My child is not currently attending school (e.g., homeschooling)
- ◯ My child does not need any supports at school
- ◯ My child needs but is not receiving supports at school
- ◯ My child is receiving supports at school
- ◯ Prefer not to answer

Please provide any additional comments:

____

Please describe what supports and why

____

		Strongly Disagree	Disagree	Somewhat Disagree	Neither Agree Nor Disagree	Somewhat Agree	Agree	Strongly Agree	Not applicable	Prefer not to answer
a	How much do you agree with the following.... My child(ren) receives an appropriate TYPES of support at school that meets their needs (e.g., access to EA, resource teacher, nursing, etc.)	◯	◯	◯	◯	◯	◯	◯	◯	◯
b	I am satisfied with the QUALITY of supports my child(ren) receives at school	◯	◯	◯	◯	◯	◯	◯	◯	◯
c	My child receives the appropriate AMOUNT of supports at school (based on their needs)	◯	◯	◯	◯	◯	◯	◯	◯	◯

Other Supports

4 What services and supports has your child received and/or taken part in during the past 3 years? Check all that apply.

- ☐ Medical services received at clinics (e.g., doctors, surgeons)
- ☐ Respite worker
- ☐ Childcare
- ☐ Other
- ☐ My child hasn't received any additional supports in the past 3 years
- ☐ Prefer not to answer

Please describe:

____

5 Tell us more about community-based (outside of school) health-related supports and therapies (e.g., occupational therapy, physiotherapy) your child has received in the past 3 years (please check as many as applicable).

- ◯ My child does not need any community-based health-related supports and therapies
- ◯ My child needs but is not receiving community-based health-related supports and therapies
- ◯ My child is receiving community-based supports
- ◯ Prefer not to answer

Please describe why

____

		Strongly Disagree	Disagree	Somewhat Disagree	Neither Agree Nor Disagree	Somewhat Agree	Agree	Strongly Agree	Not applicable	Prefer not to answer
a	How much do you agree with the following... Overall, my child has access to appropriate TYPES of community-based health-related supports and therapies they need (i.e. community aide, allied health therapists)	○	○	○	○	○	○	○	○	○
b	Overall, my child receives the appropriate AMOUNT of community-based health-related supports and therapies	○	○	○	○	○	○	○	○	○
c	Overall, I am satisfied with the QUALITY of community-based health-related supports and therapies that my child receives	○	○	○	○	○	○	○	○	○

6 Tell us more about medical services and caregiving supports (e.g., doctors' appointments, respite worker) your child has received in the past 3 years.

- ○ My child does not need any medical services and caregiving supports
- ○ My child needs but is not receiving medical services and caregiving supports
- ○ My child is receiving medical services and caregiving supports
- ○ Prefer not to answer

Please describe why

____

		Strongly Disagree	Disagree	Somewhat Disagree	Neither Agree Nor Disagree	Somewhat Agree	Agree	Strongly Agree	Not applicable	Prefer not to answer
a	How much do you agree with the following... Overall, my child has access to appropriate TYPES of medical services and/or caregiving supports they need (i.e. doctors, specialists, nurse practitioners, care coordinators	○	○	○	○	○	○	○	○	○
b										

	Overall, my child receives the appropriate AMOUNT of medical services and/or caregiving supports	◯	◯	◯	◯	◯	◯	◯	◯	◯
c	Overall, I am satisfied with the QUALITY of medical services and/or caregiving supports that my child receives	◯	◯	◯	◯	◯	◯	◯	◯	◯

7 Tell us more about recreational activities (e.g., swimming, drama) your child attended in the past 3 years.

- ◯ My child is not interested in participating in any recreational activities
- ◯ My child wants/needs to participate in recreational activities but they are not available in my area
- ◯ My child wants/needs to participate in recreational activities but they are not inclusive or it is impossible for my child to participate in them
- ◯ My child is participating in recreational activities
- ◯ Prefer not to answer

		Strongly Disagree	Disagree	Somewhat Disagree	Neither Agree Nor Disagree	Somewhat Agree	Agree	Strongly Agree	Not applicable	Prefer not to answer
a	How much do you agree with the following... Overall, my child has access to appropriate TYPES of recreational activities they need (i.e. camps, special Olympics)	◯	◯	◯	◯	◯	◯	◯	◯	◯
b	Overall, my child receives the appropriate AMOUNT of recreational activities they are interested in and can participate in	◯	◯	◯	◯	◯	◯	◯	◯	◯
c	Overall, I am satisfied with the QUALITY of recreational activities in which my child participates	◯	◯	◯	◯	◯	◯	◯	◯	◯

8 Tell us more about parental and family supports for siblings, parents, caregivers (for example, individual/family therapy, parental support group) that you and/or your family members participated in during the past 3 years.

- ◯ My family does not need any parental and family supports
- ◯ My family needs but is not receiving
- ◯ My family is receiving parental and family supports
- ◯ Prefer not to answer

Please describe why: ______________________________

		Strongly Disagree	Disagree	Somewhat Disagree	Neither Agree Nor Disagree	Somewhat Agree	Agree	Strongly Agree	Not applicable	Prefer not to answer
a	How much do you agree with the following... Overall, my family has access to appropriate TYPES of parental and family supports we need (i.e. respite, community workers)	○	○	○	○	○	○	○	○	○
b	Overall, my family receives the appropriate AMOUNT of parental and family supports we receive	○	○	○	○	○	○	○	○	○
c	Overall, I am satisfied with the QUALITY of parental and family supports our family receives	○	○	○	○	○	○	○	○	○

9 Have any of the services or supports that your child(ren) and/or family receive been interrupted because of the pandemic?

- ○ Yes
- ○ No
- ○ Prefer not to answer

Which ones? Please select all that apply.

- ☐ School-based supports
- ☐ Therapies inside of school
- ☐ Therapies outside of school
- ☐ Medical services (e.g., surgeries, diagnostic appointments)
- ☐ Mental health supports
- ☐ Home-based supports (respite, nursing care)
- ☐ Social work and/or care coordination
- ☐ Recreational activities
- ☐ Parental and family supports
- ☐ Financial support
- ☐ Disability supports
- ☐ Prefer not to answer

Have these services since resumed in a way that works for your child and family?

- ○ Yes
- ○ No

Please provide any additional comments if you wish

____

10 Did your child/family apply for any governmental financial support in the last three years?

- ○ Yes
- ○ No

Did your child/family receive any governmental financial support in the last three years?

- ○ Yes
- ○ No

		Strongly Disagree	Disagree	Somewhat Disagree	Neither Agree Nor Disagree	Somewhat Agree	Agree	Strongly Agree	Not applicable	Prefer not to answer
a	How much do you agree with the following?.. Governmental financial support that my child/family received was enough to cover their healthcare needs and supports they receive.	○	○	○	○	○	○	○	○	○
b	I was able to spend this financial support in the way that worked best for my family	○	○	○	○	○	○	○	○	○

Please add any comments

____

11 What other supports do your child and family need to be at their best?

____

12 Overall, how happy/satisfied is your child with the following?

		Completely dissatisfied	Mostly dissatisfied	Somewhat dissatisfied	Neither satisfied nor dissatisfied	Somewhat satisfied	Mostly satisfied	Completely satisfied	Not applicable	Prefer not to answer
a	Having friendships and close connections (with those who are important to them)	○	○	○	○	○	○	○	○	○
b	School and education	○	○	○	○	○	○	○	○	○
c	Family life and relationships, connecting with family members	○	○	○	○	○	○	○	○	○
d	Opportunities to take part in activities that they enjoy	○	○	○	○	○	○	○	○	○
e	Opportunities to engage in activities to move their bodies	○	○	○	○	○	○	○	○	○
f	Looking forward to the future (events, school, adolescence/adulthood)	○	○	○	○	○	○	○	○	○
g	Feeling accepted and included in their everyday life	○	○	○	○	○	○	○	○	○

13 Has anything changed during Covid that you want to continue with regard to the services and supports that your child and family receive? Think about frequency, mode of delivery, etc. Please select all that apply.

- ☐ Virtual/hybrid learning environment
- ☐ Virtual appointments for non-urgent needs (for services or healthcare)
- ☐ Being at home, spending time as a family
- ☐ Other
- ☐ Prefer not to answer

Please describe

____

14 How were your child(ren) impacted by the following during Covid?

		extremely negative	negative	somewhat negative	neutral	somewhat positive	positive	extremely positive	not applicable	prefer not to answer
a	Virtual/hybrid learning environment	◯	◯	◯	◯	◯	◯	◯	◯	◯
b	Virtual appointments for healthcare	◯	◯	◯	◯	◯	◯	◯	◯	◯
c	Virtual appointments for therapies	◯	◯	◯	◯	◯	◯	◯	◯	◯
d	Virtual recreation activities	◯	◯	◯	◯	◯	◯	◯	◯	◯
e	Being at home, spending time as a family	◯	◯	◯	◯	◯	◯	◯	◯	◯
f	Parents' support with schooling	◯	◯	◯	◯	◯	◯	◯	◯	◯
g	Parents' support with therapy, learning new skills	◯	◯	◯	◯	◯	◯	◯	◯	◯
h	Virtually connecting with friends or family	◯	◯	◯	◯	◯	◯	◯	◯	◯

15 How much do you agree with the following statement: There is clear communication and coordination between services and supports my child receives in different areas (e.g., healthcare, supports at schools, social supports)?

- ◯ Disagree
- ◯ Somewhat Disagree
- ◯ Neither Agree Nor Disagree
- ◯ Somewhat Agree
- ◯ Agree
- ◯ Strongly Agree
- ◯ Not applicable
- ◯ Prefer not to answer

16 Have you received help in finding and accessing services and supports for your child?

- ◯ Yes
- ◯ No

Who has helped you with this?

- ☐ Friend or family
- ☐ Community organization
- ☐ Parent networks (online or offline)
- ☐ Formal navigator
- ☐ Other
- ☐ Prefer not to answer

Please describe

____

Overall, how happy/satisfied were you with the navigation provided?

- ◯ not at all
- ◯ happy/satisfied to a very small extent
- ◯ happy/satisfied to a small extent
- ◯ happy/satisfied to a moderate extent
- ◯ happy/satisfied to a fairly great extent
- ◯ happy/satisfied to a great extent
- ◯ happy/satisfied to a very great extent
- ◯ not applicable
- ◯ prefer not to answer

17 Moving forward, how important are the following for you and your child

		not at all	important to a very small extent	important to a small extent	important to a moderate extent	important to a fairly great extent	important to a great extent	important to a very great extent	not applicable	prefer not to answer
a	Continuous services (no aging out)	◯	◯	◯	◯	◯	◯	◯	◯	◯
b	Service providers who are regular and don't change all the time	◯	◯	◯	◯	◯	◯	◯	◯	◯
c	Service providers who are a good fit for your family and have some training or capacity to learn about my child	◯	◯	◯	◯	◯	◯	◯	◯	◯
d	School and healthcare systems that communicate with each other	◯	◯	◯	◯	◯	◯	◯	◯	◯
e	Services and therapies that are easy to navigate	◯	◯	◯	◯	◯	◯	◯	◯	◯
f	Services and therapies that are available in my area	◯	◯	◯	◯	◯	◯	◯	◯	◯
g	Services and therapies that are affordable or covered by disability supports	◯	◯	◯	◯	◯	◯	◯	◯	◯
h	Information about services that is easy to find and access	◯	◯	◯	◯	◯	◯	◯	◯	◯
i	Option of online and/or in-person schooling, depending on your child's needs	◯	◯	◯	◯	◯	◯	◯	◯	◯
j	Option of remote and/or in-person delivery of services and supports, depending on your child's needs	◯	◯	◯	◯	◯	◯	◯	◯	◯
k										

	Option of remote (telehealth or virtual) and/or in-person medical appointments, depending on your child's needs	○	○	○	○	○	○	○	○	○
l	Recreational activities that meet my child's needs and preferences	○	○	○	○	○	○	○	○	○
m	Virtual connections with children of the same age as my child	○	○	○	○	○	○	○	○	○
n	Virtual connections with other caregivers	○	○	○	○	○	○	○	○	○

18 Do you have any additional comments you wish to share?

____
